# Extensive secondary contact among three glacial lineages of Arctic Char (*Salvelinus alpinus*) in Labrador and Newfoundland

**DOI:** 10.1002/ece3.4893

**Published:** 2019-01-28

**Authors:** Sarah J. Salisbury, Gregory R. McCracken, Donald Keefe, Robert Perry, Daniel E. Ruzzante

**Affiliations:** ^1^ Department of Biology Dalhousie University Halifax Nova Scotia Canada; ^2^ Department of Environment and Conservation Corner Brook Newfoundland and Labrador Canada

**Keywords:** Arctic char, glacial refugia, introgression, mitochondrial DNA, North America, phylogeography, secondary contact

## Abstract

**Aim:**

The Pleistocene glaciation event prompted the allopatric divergence of multiple glacial lineages of Arctic char (*Salvelinus alpinus*), some of which have come into secondary contact upon their recolonization of the Holarctic. While three glacial lineages (Arctic, Atlantic, and Acadian) are known to have recolonized the western Atlantic, the degree of overlap of these three lineages is largely unknown. We sought to determine the distribution of these three glacial lineages in Labrador and Newfoundland at a fine spatial scale to assess their potential for introgression and their relative contribution to local fisheries.

**Location:**

Labrador and Newfoundland, Canada.

**Methods:**

We sequenced a portion of the D‐loop region in over 1,000 Arctic char (*S. alpinus*) samples from 67 locations across Labrador and Newfoundland.

**Results:**

Within Labrador, the Arctic and Atlantic lineages were widespread. Two locations (one landlocked and one with access to the sea) also contained individuals of the Acadian lineage, constituting the first record of this lineage in Labrador. Atlantic and Acadian lineage individuals were found in both eastern and western Newfoundland. Multiple sampling locations in Labrador and Newfoundland contained fish of two or more different glacial lineages, implying their introgression. Glacial lineage did not appear to dictate contemporary genetic divergence between the pale and dark morph of char present in Gander Lake, Newfoundland. Both were predominately of the Atlantic lineage, suggesting the potential for their divergence in sympatry.

**Main conclusions:**

Our study reveals Labrador and Newfoundland to be a unique junction of three glacial lineages which have likely hybridized extensively in this region.

## 1 INTRODUCTION

Glaciation events are a significant driver of evolution, physically isolating species into separate glacial refugia which can undergo allopatric divergence for thousands of years (Fraser, Nikula, Ruzzante, & Waters, 2012; Hewitt, 2000, 2004). During allopatry, populations may experience differential selection and drift resulting in the formation of genetically distinct glacial lineages (Hewitt, 2003; Moore, Bajno, Reist, & Taylor, 2015; Ruzzante et al., 2008). Retreating glaciers allowed access to new environments, sometimes facilitating secondary contact (Hewitt, 2000; Soltis, Morris, McLachlan, Manos, & Soltis, 2006; Swenson & Howard, 2005). Upon secondary contact, glacial lineages may demonstrate: (a) extensive gene flow, leading to complete genomic introgression; (b) complete reproductive isolation and an absence of gene flow; and (c) some intermediate level of gene flow (Hewitt, 1988; Noor, 1999; Schluter, 2001). The degree of hybridization is likely to depend on the accumulated genetic divergence among lineages and on the adaptive quality of each gene (Hewitt, 1988). The amount of genetic divergence accumulated between glacial lineages and the degree of erosion of this divergence in secondary contact zones can significantly influence the contemporary genetic structure of a species (Bernatchez & Wilson, 1998; Hewitt, 2000, 2004). These areas of secondary contact and hybridization therefore not only inform conservation management but also offer natural experiments for the study of the factors driving speciation (Hewitt, 1988).

Arctic char (*Salvelinus alpinus*) is one species demonstrating multiple secondary contact zones between glacial lineages which arose from allopatry during the Pleistocene (Brunner, Douglas, Osinov, Wilson, & Bernatchez, 2001; Moore et al., 2015). Five glacial lineages of Arctic char have been described based on mtDNA: Arctic, Atlantic, Acadian, Beringian, and Siberian (Brunner et al., 2001; Moore et al., 2015). Evidence for secondary contact has been observed between the Beringian and Arctic lineages in Russia and western North America (Brunner et al., 2001; Esin, Bocharova, Mugue, & Markevich, 2017; Moore et al., 2015; Oleinik, Skurikhina, & Kukhlevsky, 2017) and between the Arctic and Atlantic lineages in Nunavut and Labrador, Canada, and between the Atlantic and Acadian lineages in Newfoundland, Canada (Brunner et al., 2001; Moore et al., 2015; Salisbury et al., 2018; Wilson, Hebert, Reist, & Dempson, 1996). However, our knowledge of these secondary contacts is at a coarse spatial scale, particularly in Atlantic Canada (Brunner et al., 2001; Moore et al., 2015).

The Laurentide Ice Sheet covered this region during the Pleistocene (Bryson, Wendland, Ives, & Andrews, 1969). It retreated fully from Newfoundland between 13,000 and 9,000 years BP (Bryson et al., 1969; Dyke, 2004; Shaw et al., 2006) and from Labrador between 9,000 and 7,500 years BP (Bryson et al., 1969; Jansson, 2003; Occhietti, Parent, Lajeunesse, Robert, & Govare, 2011). The vast quantities of fresh water draining from the retreating glaciers into the Atlantic Ocean allowed anadromous Arctic char to extensively colonize Labrador and Newfoundland (Power, 2002). Some of the lakes colonized by anadromous char in Labrador subsequently lost their access to the sea resulting in contemporarily landlocked char populations (Scott & Crossman, 1998). Other anadromous char populations (particularly in Newfoundland) lost their anadromous lifestyle and remain lacustrine residents year‐round (Scott & Crossman, 1998).

The glacial lineages present in anadromous versus landlocked populations remain largely unknown in this region. Landlocked and anadromous populations might have been founded by different lineages, for example, if one was better adapted to a particular environment or life history. Alternatively, landlocked populations could have been founded only by lineages that were present before access to these lakes was lost. Investigation of which lineages are present in these two types of populations may therefore give an indication of the timing of clonization by different glacial lineages (Moore et al., 2015).

Within‐lake genetic structure, previously found in Labrador and Newfoundland char populations, may also be influenced by glacial lineage. Glacial lineage has been suggested as the origin of the substantial genetic divergence observed between a pale and a dark morph documented for Gander Lake in Newfoundland (Gomez‐Uchida, Dunphy, O'Connell, & Ruzzante, 2008). Salisbury et al. (2018) alternatively found that the genetic structure in two landlocked lakes and one sea‐accessible lake in Labrador were unrelated to glacial lineage.

Anadromous Arctic char populations are economically significant and form the basis of a commercial, recreational, and subsistence fishery in Labrador (DFO, 2001; Dempson, Shears, Furey, & Bloom, 2008). Historic allopatry may be an important underlying influence on genetic structure if char of different glacial lineages contribute to the fishery but remain reproductively isolated. While it is currently unknown which glacial lineages contribute to the Labrador fishery, this knowledge is potentially critical for its management.

Here, we investigated the consequences of secondary contact of the Arctic, Atlantic, and Acadian glacial lineages across Labrador and Newfoundland at a fine spatial scale. We predicted that the Arctic lineage would be more prevalent in northern populations than the Atlantic lineage based on the hypothesis that Labrador was colonized from the north by the Arctic lineage and from the south by the Atlantic lineage. Hybridization among lineages was expected to be prevalent and evidenced by multiple lineages co‐existing in single populations. In Labrador, we anticipated that lineages which colonized more recently would be present only in the sea‐accessible but not the landlocked populations. Finally, we hypothesized that the large divergence between the pale and dark morphs within Gander Lake was due to their founding by different glacial lineages. To test these hypotheses, we employed mtDNA to identify the glacial lineage of hundreds of fish across Labrador and Newfoundland.

## 2 MATERIALS AND METHODS

### 2.1 Sampling

Tissue samples (*N* = 1,329) were collected between 2000 and 2015 from Labrador and Newfoundland. Landlocked and sea‐accessible Labrador locations were distributed among 10 drainages (fjords or bays). The samples from three sites in Labrador (Ramah (R01), WP132 (S03), and WP133 (S04)) were used previously in Salisbury et al. (2018). Collections from western Newfoundland originate from five landlocked lakes in the Upper Humber River (see Gomez‐Uchida, Knight, & Ruzzante, 2009; Gomez‐Uchida, Palstra, Knight, & Ruzzante, 2013). Collections from eastern Newfoundland originate from two locations containing only freshwater residents: Gander Lake (including samples of the pale and dark morphs described for this lake (Gomez‐Uchida et al., 2008)) and Wing Pond.

Samples from Labrador were collected using electrofishing in the rivers (sea‐accessible sites) and variably sized standardized nylon monofilament gillnets (1.27–8.89 cm diagonal) at the landlocked and sea‐accessible lake sites. Samples were collected from anadromous char populations in the Okak and Voisey regions as well as from the Fraser River, Anaktalik River, and Tikkoatokak River. These populations contribute to the three stock complexes of the commercial Labrador char fishery (Okak, Voisey, Nain) (DFO, 2001). Gander Lake was sampled using Lundgren multimesh gillnets (Hammar & Filipsson, 1985) (bar length from 0.625 cm to 7.5 cm) (see Gomez‐Uchida et al., 2008, for more details). The Upper Humber River was sampled using fyke nets and electrofishing (see Gomez‐Uchida et al., 2009, for more details). Wing Pond was sampled with gillnets. Fish were weighed, measured for fork length (FL) in mm, and assessed for sex and maturity. Tissue samples (fin or gill) were obtained and immediately stored in 95% ethanol; alternatively, some fin clip samples were stored dry. All samples were collected in collaboration with the Department of Environment and Conservation for Labrador and Newfoundland and/or Parks Canada and in accordance with Dalhousie University's Animal Ethics Guidelines.

### 2.2 DNA extraction, amplification, and genotyping

Tissue samples were digested at 55°C for approximately eight hours using Proteinase K (Bio Basic Inc., Markham, ON, Canada). DNA was then extracted using a Multiprobe II plus liquid handling system (Perkin Elmer, Waltham, MA, USA) using a glassmilk protocol modified from Elphinstone, Hinten, Anderson and Nock (2003).

The left domain region of the mitochondrial control region was amplified and sequenced following Moore et al. (2015). In brief, the primers *Tpro2* (Brunner et al., 2001) and *SalpcrR* (Power, Power, Reist, & Bajno, 2009) were used to amplify the entire control region using the thermocycler program and PCR outlined in Brown Gladden, Postma Maiers, Carmichael, and Reist (1995). A shorter fragment was amplified using *Char3 *instead of *Tpro2 *for a minority of samples which had poor quality as determined from visual inspection of a 1% agarose gel. For all samples, a total of ~500 bp of the left domain was sequenced using *Char3* (Power et al., 2009) at MacrogenUSA (Rockville, MD). Each unique haplotype detected was validated by resequencing a representative sample for each haplotype using *Tpro2*.

### 2.3 Analyses

Our sequences were trimmed, validated, and aligned using GENEIOUS (10.0.9, Auckland, NZ, www.geneious.com). Using default alignment parameters, our sequences were aligned to a reference haplotype set (control region haplotypes verified by Moore et al. (2015) and Salisbury et al. (2018)), and control region sequences for three other salmonid species present in the region (brook trout (*Salvelinus fontinalis*), lake trout (*Salvelinus namaycush*), and Atlantic salmon (*Salmo salar*); for accession numbers see Supporting Information Table S1). Sequences verified as Arctic char were ascribed to the reference haplotype(s) for which they had 0 basepair differences. Non‐char sequences were ascribed to the brook trout, lake trout, or Atlantic salmon haplotype to which they had the minimum number of basepair differences.

A representative forward sequence for each unique haplotype (i.e., those sequences which contained one or more basepair differences from those haplotypes verified by Moore et al. (2015)) was aligned with its reverse complement (sequenced with *Tpro2*) using a pairwise GENEIOUS alignment and default parameters to create a consensus sequence. The consensus sequences for these unique haplotypes were then aligned with the reference haplotype set using a GENIOUS alignment. A gap penalty of 7 was used, and all other parameters were kept at default values. A maximum‐likelihood tree was constructed based on this alignment using the PhyML (Guindon & Gascuel, 2003) plugin in GENEIOUS to compare the phylogenetic relationships among these unique consensus sequences with those haplotypes verified by Moore et al. (2015) and those of an outgroup species (brook trout). The Nearest Neighbour Interchange topology search algorithm and the HKY85 + I + G model were used to calculate 1,000 bootstraps for each node following Moore et al. (2015).

A haplotype map based on all unique haplotypes found in this study along with all haplotypes verified by Moore et al. (2015) was created using PopArt version 1.7 (Leigh & Bryant, 2015). Haplotypes were trimmed to 501 bp, the length for which all haplotypes had no missing base pairs, since PopArt masks missing base pairs. This meant that haplotype ATL04 and a unique haplotype ATL31 were indistinguishable in this analysis since the SNP differentiating these haplotypes lies outside of this 501 bp region. The haplotype map was created using a Median‐Joining network (Bandelt, Forster, & Röhl, 1999) with an Epsilon value of 0.

A spatial analysis of molecular variance (SAMOVA 2.0) (Dupanloup, Schneider, & Excoffier, 2002) was employed to detect groups of sampling locations whose *F*_CT_ were maximally differentiated based on mtDNA sequences. All sequences were aligned using GENEIOUS alignment and default parameters and trimmed to 482 bp to include all relevant SNPs differentiating haplotypes. Sampling locations with fewer than 10 sequences were excluded from the analysis to minimize the probability of biased groupings due to small sampling size. *F*_CT_ values were estimated using a simulated annealing optimization process for *K* = 2–10 groups for all sampling locations and for only the Labrador sampling locations, and for *K* = 2–4 groups for only the Newfoundland sampling locations. For each *K*‐value, molecular distance was calculated using Tamura and Nei distance between all sampling locations and between only those sampling locations connected using a Delaunay network (Delaunay, 1934) based on the latitude and longitude of each sampling location. The use of a Delaunay network limits groupings to geographically proximate sampling locations. Simulations were run for 10,000 steps from 100 initial configurations using a missing data value of 1 (such that the entire 482 bp was included in the analysis).

Linear regressions between latitude and the number of lineages present in each location as well as binomial logistic regressions of latitude on the presence or absence (coded as 1 and 0, respectively) of each of the relevant glacial lineages were conducted using R (R Core Team, 2013) for all locations, only Labrador locations, and only Newfoundland locations.

## 3 RESULTS

### 3.1 Species distribution

Samples from 59 locations in Labrador (of which 43 are sea‐accessible and 16 are landlocked (Anderson, 1985)) and eight locations in Newfoundland (all containing lacustrine residents) for a total of 67 locations overall were successfully sequenced (Table 1). Five locations in Labrador were excluded from analyses due to poor sequence quality (*N* = 109 individuals). A further 20 individuals from across the remaining 67 locations were excluded from analyses due to poor sequence quality. A total of *N* = 1,296 individuals were successfully sequenced across all locations in Labrador and Newfoundland (Table 1). Of these, 1,133 had haplotypes consistent with the Arctic char species. The remaining 163 individuals were identified as brook trout, lake trout, or Atlantic salmon. All locations contained at least one Arctic char haplotype except G06 which only contained Atlantic salmon. In the remaining locations, we sampled between 1 and 48 Arctic char (median = 18.5), which made up between 2% and 100% of the haplotypes in each sample.

**Table 1:** Number of Arctic char (*Salvelinus alpinus*) samples, glacial lineages, and haplotypes as well as number of brook trout (*Salvelinus fontinalis*), lake trout (*Salvelinus namaycush*), and Atlantic salmon (*Salmo salar*) samples verified by mtDNA sequencing at sampling locations across Labrador and Newfoundland. Accessibility of locations (A for sea‐accessible, L for landlocked)

Site	Drainage	Watershed	Latitude, longitude	Access	Number of *S. fontinalis*	Number of *S. namaycush*	Number of *S. Salar*	Number of *S. alpinus*	Number of *S. alpinus* lineages	Number of *S. alpinus* haplotypes
N01	Nachvak	Schooner	59°05'47.50, −63°30'33.58	A				15	2	3
N02	Nachvak	Palmer River	58°56'59.60, −63°52'55.35	A				24	2	2
N03	Nachvak	McCormick's River	59°01'00.94, −63°44'39.15	A				24	2	3
N04	Nachvak	McCormick's River	59°00'28.61, −63°44'16.21	A				19	2	2
R01	Ramah	Stecker River	58°50'28.96, −63°28'38.66	A				48	2	4
S01	Saglek	North Arm Brook	58°32'53.92, −63°27'38.09	A				25	2	2
S02	Saglek	Southwest Arm Brook	58°29'07.27, −63°27'47.04	A				24	2	4
S03	Saglek	Southwest Arm Brook	58°16'48.58, −63°58'09.47	L				24	1	1
S04	Saglek	Southwest Arm Brook	58°16'18.02, −64°01'52.90	L				24	1	3
S05	Saglek	Pangertok Inlet River	58°19'46.64, −63°11'05.05	A				14	2	3
S06	Saglek	Kiyuktok Brook	58°26'55.46, −62°48'36.36	A				23	1	1
H02	Hebron	Ikarut River	58°09'17.07, −63°06'10.56	A				23	2	3
H03	Hebron	Ikarut River	58°10'25.72, −63°17'37.74	A	8			16	2	3
H04	Hebron	Ikarut River	58°08'46.00, −63°35'28.77	L				24	1	1
H05	Hebron	Hebron	58°03'42.64, −63°12'54.67	A				4	2	2
H07	Hebron	River 105 (Unnamed)	58°05'10.83, −63°43'50.18	A				24	2	2
H09	Hebron	River 104 (Unnamed)	57°56'11.77, −63°28'31.80	A				24	2	2
H10	Hebron	River 104 (Unnamed)	57°51'57.96, −63°32'22.08	A	2			22	2	2
H11	Hebron	River 104 (Unnamed)	57°50'20.23, −63°32'20.79	A				21	2	3
H12	Hebron	River 104 (Unnamed)	57°46'29.73, −63°36'50.69	A				3	2	2
H13	Hebron	Unnamed River	57°58'16.60, −63°12'56.64	A				24	2	2
H14	Hebron	River 103 (Unnamed)	58°02'23.19, −63°01'55.97	A				23	2	3
H15	Hebron	River 103 (Unnamed)	58°00'37.93, −63°02'25.85	A	3			20	2	2
H16	Hebron	River 103 (Unnamed)	57°44'37.87, −63°21'10.96	A				18	2	2
K01	Okak	Siugak Brook	57°37'02.94, −62°10'46.77	A	11		6	7	2	2
K02	Okak	Siugak Brook	57°36'07.39, −62°25'25.77	A	26			6	1	1
K03	Okak	Siugak Brook	57°43'35.33, −62°28'24.10	A				24	1	1
K04	Okak	Siugak Brook	57°39'41.79, −62°57'16.01	L				21	1	2
K05	Okak	North River	57°30'05.72, −62°44'35.43	A				24	2	3
K06	Okak	North River	57°38'20.95, −63°13'58.52	L				24	1	1

Site	Drainage	Watershed	Latitude, Longitude	Access	*S. fontinalis*	*S. namaycush*	*S. Salar*	*S. alpinus*	Number of lineages	Number of haplotypes
T01	Tikkoatokak	Kingurutik River	56°52'48.33, −62°37'19.58	A				24	2	5
T02	Tikkoatokak	Kingurutik River	57°08'55.83, −62°52'41.37	A				26	1	2
T03	Tikkoatokak	Kingurutik River	57°17'08.88, −63°42'48.22	A		1		7	1	1
T04	Tikkoatokak	Kamanatsuk Brook	56°44'19.87, −62°33'48.41	A	3			21	2	2
T05	Tikkoatokak	Kamanatsuk Brook	56°45'26.58, −62°52'14.21	L	55			1	1	1
F01	Nain	Fraser	56°41'22.74, −63°27'56.10	A				24	2	4
A01	Anaktalik	Anaktalik	56°29'52.47, −62°55'46.86	A				24	2	3
A02	Anaktalik	Anaktalik	56°34'53.08, −63°19'24.07	L		3		22	1	1
V01	Voisey	Kogluktokoluk Brook	56°18'47.06, −62°10'07.13	A	2			22	2	2
V02	Voisey	Kogluktokoluk Brook	56°17'38.10, −62°16'56.08	A				2	2	2
V03	Voisey	Kogluktokoluk Brook	56°17'38.28, −62°23'34.38	A	6			18	2	2
V04	Voisey	Kogluktokoluk Brook	56°16'41.35, −62°25'05.54	A	10			14	2	2
V05	Voisey	Kogaluk River	56°10'13.46, −61°44'40.35	A				1	1	1
V06	Voisey	Kogaluk River	56°09'32.52, −61°45'45.79	A	7			5	1	1
V07	Voisey	Kogaluk River	56°22'08.70, −63°29'30.50	L				14	2	2
V09	Voisey	Kogaluk River	56°40'31.70, −64°00'07.50	L				9	1	1
V10	Voisey	Kogaluk River	56°36'13.70, −63°52'09.10	L				12	2	2
V11	Voisey	Kogaluk River	56°24'56.20, −64°06'08.10	L		1		23	2	2
V13	Voisey	Kogaluk River	56°09'09.70, −63°56'21.20	L				6	1	1
V14	Voisey	Kogaluk River	56°06'38.40, −63°23'18.90	L				17	1	1
V15	Voisey	Kogaluk River	56°02'52.10, −63°35'54.40	L				13	2	2
V16	Voisey	Kogaluk River	55°55'26.19, −63°08'34.22	L				7	2	2
W01	Notakwanon	Notakwanon	55°58'22.05, −61°45'15.82	A	1			3	1	1
W02	Notakwanon	Notakwanon	55°56'00.23, −62°04'15.16	A	6		4	12	1	1
W03	Notakwanon	Notakwanon	55°54'09.81, −62°06'46.41	A	2			8	3	3
W04	Notakwanon	Notakwanon	55°57'55.29, −62°19'28.48	A	1			16	2	2
W05	Notakwanon	Notakwanon	55°54'18.34, −62°59'10.25	A				12	2	2
W06	Notakwanon	Notakwanon	55°49'25.42, −63°03'34.33	A				12	2	2
W09	Notakwanon	Notakwanon	55°23'38.23, −63°16'56.28	L				16	1	1
G01	Rocky Harbour	Rocky Harbour	49°39'39.16, −57°37'31.53	L				4	1	1
G02	Rocky Harbour	Rocky Harbour	49°38'35.39, −57°33'03.40	L				6	2	2
G03	Rocky Harbour	Rocky Harbour	49°37'58.56, −57°35'16.52	L				16	2	4
G04	Rocky Harbour	Rocky Harbour	49°37'39.10, −57°36'49.91	L				12	2	4
G05	Rocky Harbour	Rocky Harbour	49°37'46.22, −57°37'40.93	L				21	1	4
G06	Rocky Harbour	Rocky Harbour	49°37'37.35, −57°41'44.16	L			5			
I01	Eastern Island	Gander	48°56'19.82, −54°41'04.31	L^a^				22	1	2
I02	Eastern Island	Gander	48°56'19.82, −54°41'04.31	L^a^				21	2	2
I03	Eastern Island	Wing	48°59'37.79, −54°09'01.97	L^a^				24	1	1
					143	5	15	1,133		

a Gander Lake and Wing Pond maintain sea access but contain lacustrine residents, and these lakes were therefore categorized as “landlocked” for analyses.

### 3.2 Glacial lineage distribution

**Figure 1:**
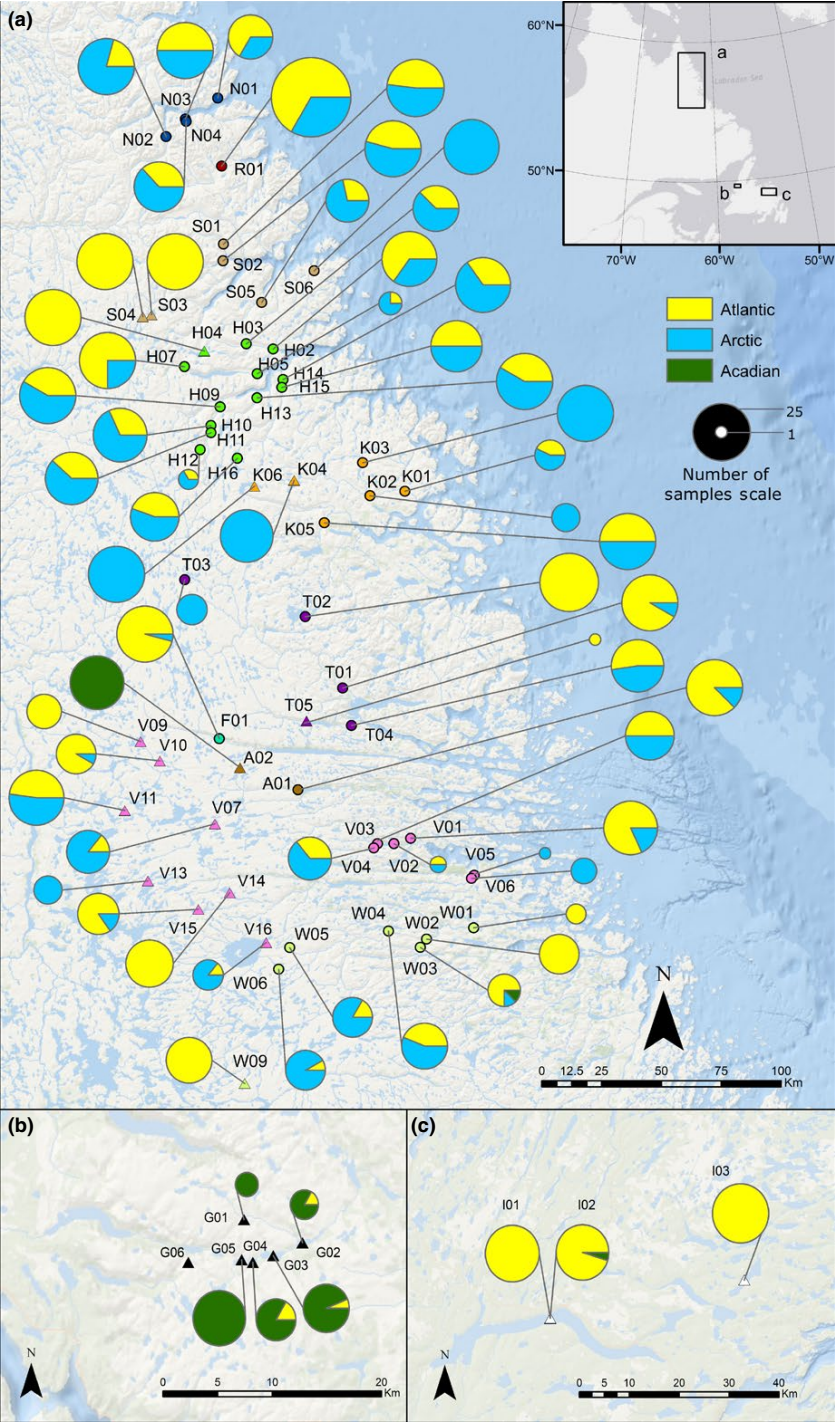
Map of sampling locations for Arctic char (*Salvelinus alpinus*) in (a) Labrador and the (b) west and (c) east coasts of Newfoundland. Sea‐accessible sites are denoted by circles, and landlocked sites are denoted by triangles. Sites of the same color are in the same drainage. Pie charts indicate the proportion of samples of the Acadian, Atlantic, or Arctic lineage observed at a given site and are scaled by sample size. Map created using ArcGIS (ESRI)

The Arctic and Atlantic glacial lineages were ubiquitous across Labrador, and both lineages were present in all 10 drainages (Figure 1a). The Arctic and Atlantic lineages were detected in 49 and 48 locations, respectively, and they co‐occurred in 39 locations. The Acadian lineage was detected in only two sampling locations in Labrador. In one landlocked location (A02, Figure 1a), all char samples were of the Acadian lineage. The second location was sea‐accessible (W03, Figure 1a), and it contained one individual of the Acadian lineage among one Arctic lineage and six Atlantic lineage individuals.

Only the Atlantic and Acadian lineages were detected in Newfoundland (Figure 1b,c). The Atlantic lineage was detected in 3/5 locations in western Newfoundland and both locations in eastern Newfoundland. The Acadian lineage was detected in all five locations of western Newfoundland, but only a single Acadian lineage individual was detected in a pale morph char from Gander Lake in eastern Newfoundland.

The overlap in the distributions of these three lineages in Labrador is suggested by a lack of correlation between latitude and the number of lineages present in each sampling location across all locations ($R_{\left(64\right)}^{2}$ = 0.036, *p* ≥ 0.12), in only Labrador sites ($R_{\left(57\right)}^{2}$ = 0.015, *p* ≥ 0.36), and in only Newfoundland sites ($R_{\left(5\right)}^{2}$ = 0.29, *p* ≥ 0.21). Binomial logistic regressions of latitude on the presence or absence (coded as 1 and 0, respectively) of the Arctic and Atlantic lineages in each Labrador sampling location were not significant (*p* ≥ 0.699, *p* ≥ 0.145, respectively). Similarly, there was no significant relationship between latitude and the presence of the Atlantic and Acadian lineages in Newfoundland sites (*p* ≥ 0.38, *p* ≥ 0.350, respectively). Across all sampling locations, the presence of the Atlantic lineage was unrelated with latitude (*p* ≥ 0.435). However, across Labrador and Newfoundland, the probability of Arctic lineage presence increased with latitude (*p* ≤ 6.46 × 10^−3^). Similarly, the presence of the Acadian lineage was inversely related to latitude (*p* ≤ 1.08 × 10^−4^).

### 3.3 Haplotype distribution

The most common haplotype within a lineage coincided with the most common haplotypes reported in Moore et al. (2015) (haplotypes at each location: see Supporting Information Table S2). A total of 86% of Atlantic lineage individuals exhibited haplotype ATL01, while 78% of Acadian lineage individuals exhibited haplotype ACD9. All Labrador samples of the Acadian lineage had this haplotype. Lastly, over 99% of Arctic lineage individuals exhibited haplotypes ARC19 or ARC24. These two haplotypes were distinguished by a single SNP outside of the region sequenced using the *Char3* primer. However, the reverse complement of 29 samples from 28 locations and nine drainages with either the ARC19 or ARC24 haplotype was sequenced using *Tpro2* and all were found to have the haplotype ARC19. Therefore, unambiguously ARC19 sequences were grouped with sequences that could be either ARC19 or ARC24 when counting the number of haplotypes present in a given site. The ARC19, ATL01, and ACD9 haplotypes were found across the modern distributions of the Arctic, Atlantic, and Acadian lineages, respectively, by Moore et al. (2015).

Other detected haplotypes which had been previously described by Moore et al. (2015) and Salisbury et al. (2018) include ACD11, ARC20, ARC22, ATL19, ATL23, ATL24, and ATL25. A single sample had the ATL19 haplotype, a dark morph char from Gander. This was also the only Atlantic haplotype other than ATL01 detected in Newfoundland. The ATL23, ATL24, and ATL25 haplotypes were only observed in S03 and S04 as described in Salisbury et al. (2018) except for one individual with ATL23 found in S02. This individual may have been washed downstream from the immediately upstream landlocked S03 and S04. This is supported by its identification as a putative migrant from S03 based on GENECLASS2 (Piry et al., 2004) results as reported in Salisbury et al. (2018).

Five samples from three landlocked sites in the Voisey drainage had shortened sequences that prevented their differentiation between ATL01 and ATL04. Since these sites also contained individuals unambiguously identified as ATL01, these shortened sequences were considered to be ATL01 when counting the number of haplotypes present in these lakes. The ATL04 haplotype was also found in 12 sea‐accessible sampling locations across seven drainages in Labrador. The reverse complement of nine of these samples from six drainages was sequenced using *Tpro2* and all were found to contain a consistent SNP in the consensus sequence, differentiating this haplotype from ATL04. One of these samples was from R01, previously mistakenly identified as ATL04 in Salisbury et al. (2018). Given the consistency of this SNP across samples from multiple drainages, sampling locations, and studies, we denoted this as a new haplotype ATL31. We considered all ATL04 haplotypes and verified ATL31 haplotypes to be a single haplotype when counting the number of haplotypes present in the 12 sea‐accessible sampling locations where these haplotypes were observed.

**Figure 2:**
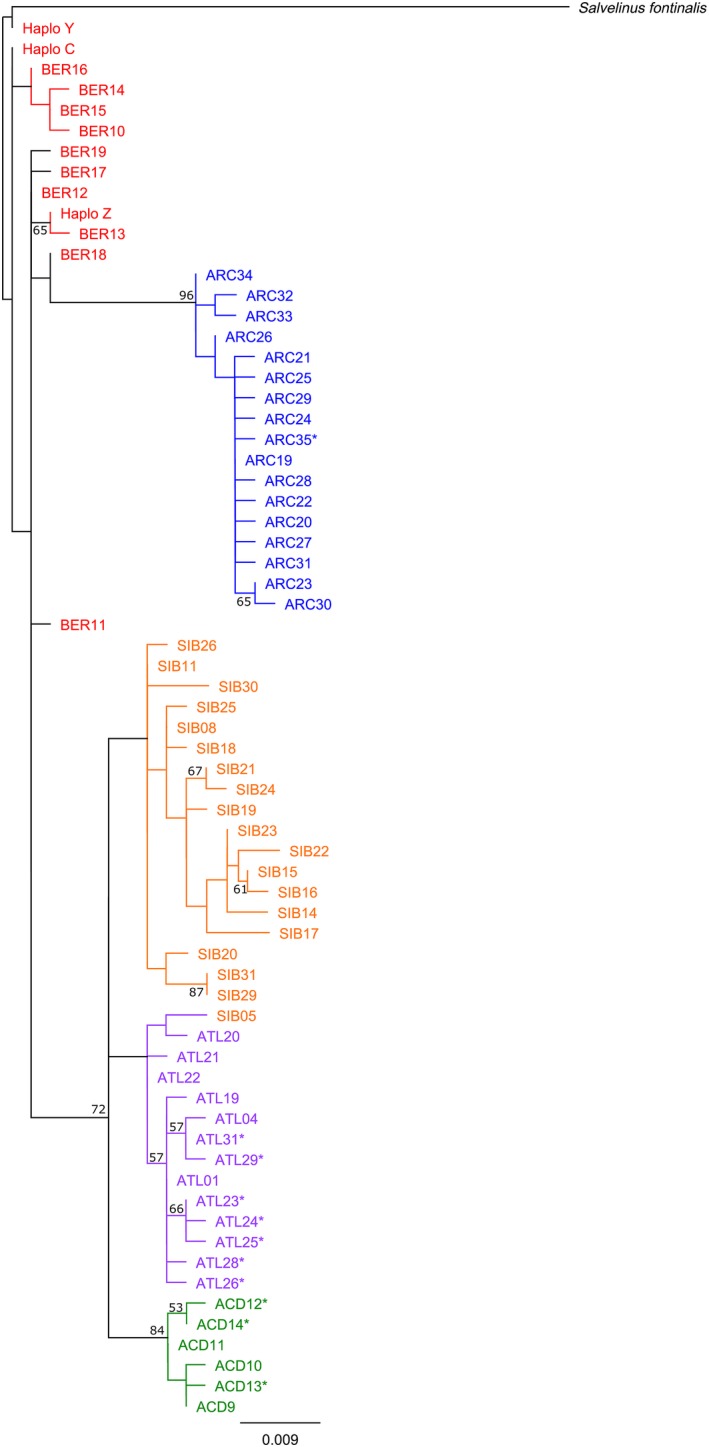
Maximum‐likelihood phylogenetic tree of Arctic char (*Salvelinus alpinus*) haplotypes of the mtDNA control region. Tree was generated using PhyML (Guindon & Gascuel, 2003) with 1,000 bootstrap replicates. Those bootstrap values greater than 50% are shown on the tree. Haplotypes are color‐coordinated by lineage as designated in Moore et al. (2015): blue—Arctic, red—Bering, orange—Siberia, purple—Atlantic, green,—Acadian. New haplotypes identified in this study and Salisbury et al., 2018 are starred

**Figure 3:**
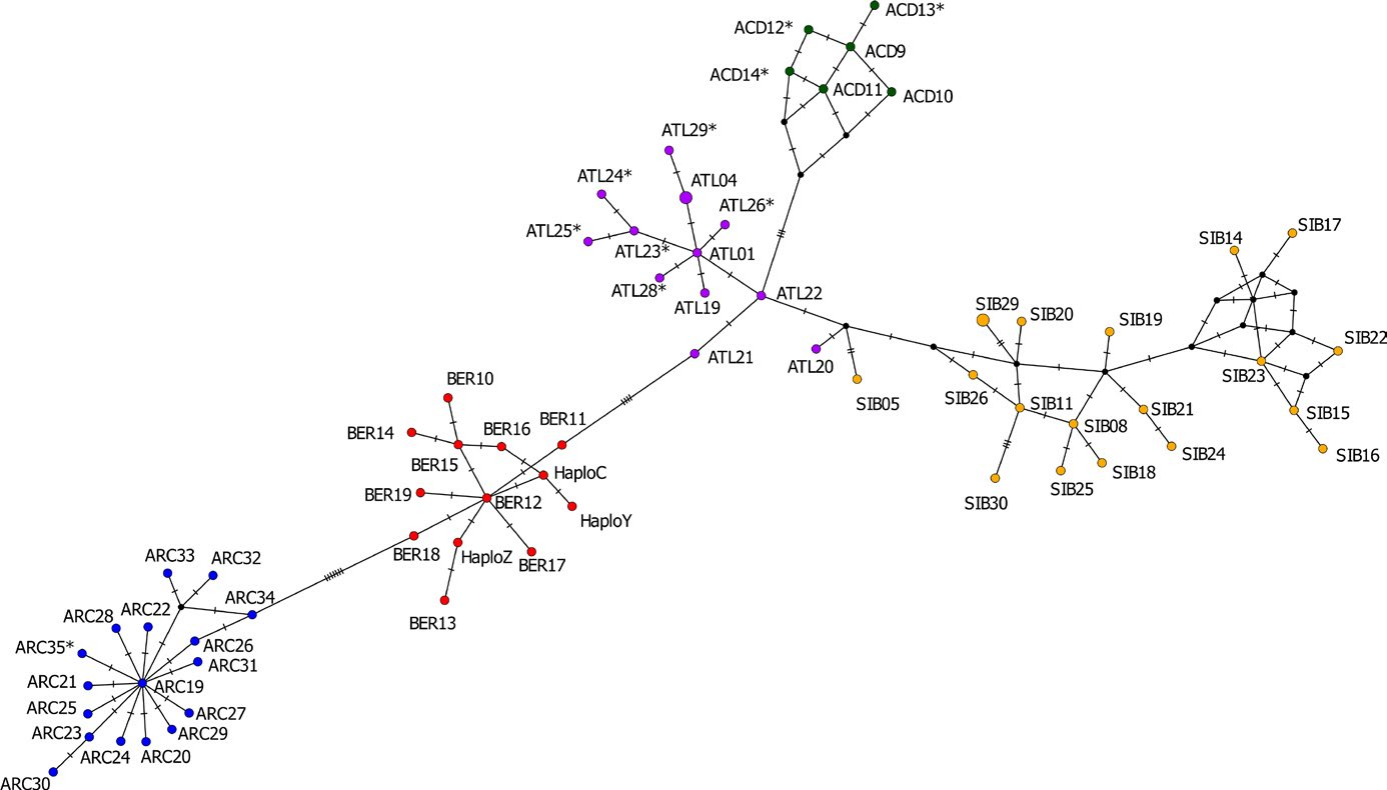
Haplotype map of Arctic char (*Salvelinus alpinus*) haplotypes created with PopArt version 1.7 (Leigh & Bryant, 2015) using a Median‐Joining network (Bandelt et al., 1999) and an Epsilon value of 0. New haplotypes identified in this study and Salisbury et al., 2018 are starred

Including ATL31, there were eight haplotypes not previously identified by Moore et al. (2015) or Salisbury et al. (2018) (Accession Numbers: MK208868–MK208871, MK208875–MK208878) (Figure 2). All new haplotypes were one base pair different from another haplotype verified by Moore et al. (2015) within their assigned lineage (Figure 3). These include three Acadian haplotypes (ACD12, ACD13, and ACD14) only observed in western Newfoundland. Four new Atlantic haplotypes were identified (ATL26, ATL28, ATL29, and ATL31). ATL26 was found in only one individual in A01. ATL28 was found in three individuals, one in F01, one in T01, and one in T02. ATL29 was found in one individual in N01. Only one new Arctic haplotype, ARC35, was observed in a single individual in T01. All new haplotypes except ATL31 were found to be at least 1 base pair different from the top hit when compared with the NCBI nr/nt database using the Megablast algorithm. ATL31 was found to have 100% identity with an Arctic char sample (Accession Number: KY122252) collected from Lake Sitasjaure, Sweden (Oleinik et al., 2017).

### 3.4 Landlocked versus sea‐accessible sampling locations

The average number of lineages observed in anadromous sites was higher than in landlocked sites (average of 1.8 lineages for anadromous sites vs. 1.3 lineages for landlocked sites, *T*_(42)_ = 3.78, *p* ≤ 0.001). Similarly, the average number of haplotypes observed in anadromous sites was higher than in landlocked sites (average of 2.3 haplotypes for anadromous sites vs. 1.8 haplotypes for landlocked sites, *T*_(45)_ = 1.96, *p* ≤ 0.056). (Note: the Gander Lake morphs (I01/I02) and Wing Pond were grouped with the landlocked lakes despite both lakes having access to the sea because their char are lacustrine residents). This effect was even more extreme when considering only Labrador sites where the corresponding average numbers were 2.3 and 1.5 haplotypes in anadromous and landlocked sites, respectively (*T*_(40)_ = 3.56, *p* ≤ 0.001). Anadromous sites in Labrador also had an average of 1.8 lineages per site, significantly more than the 1.3 lineages observed in landlocked sites (*T*_(26)_ = 3.63, *p* ≤ 0.0012).

### 3.5 SAMOVA

SAMOVA results were similar across all samples and when considering Labrador and Newfoundland sampling locations separately. Results were also similar with and without the use of a Delaunay network to take into account geographic proximity of locations. For brevity, we report only the SAMOVA results when considering all sampling locations and a Delaunay network (for results of all other SAMOVA analyses see Supporting information Figures S1 and S2).

**Figure 4:**
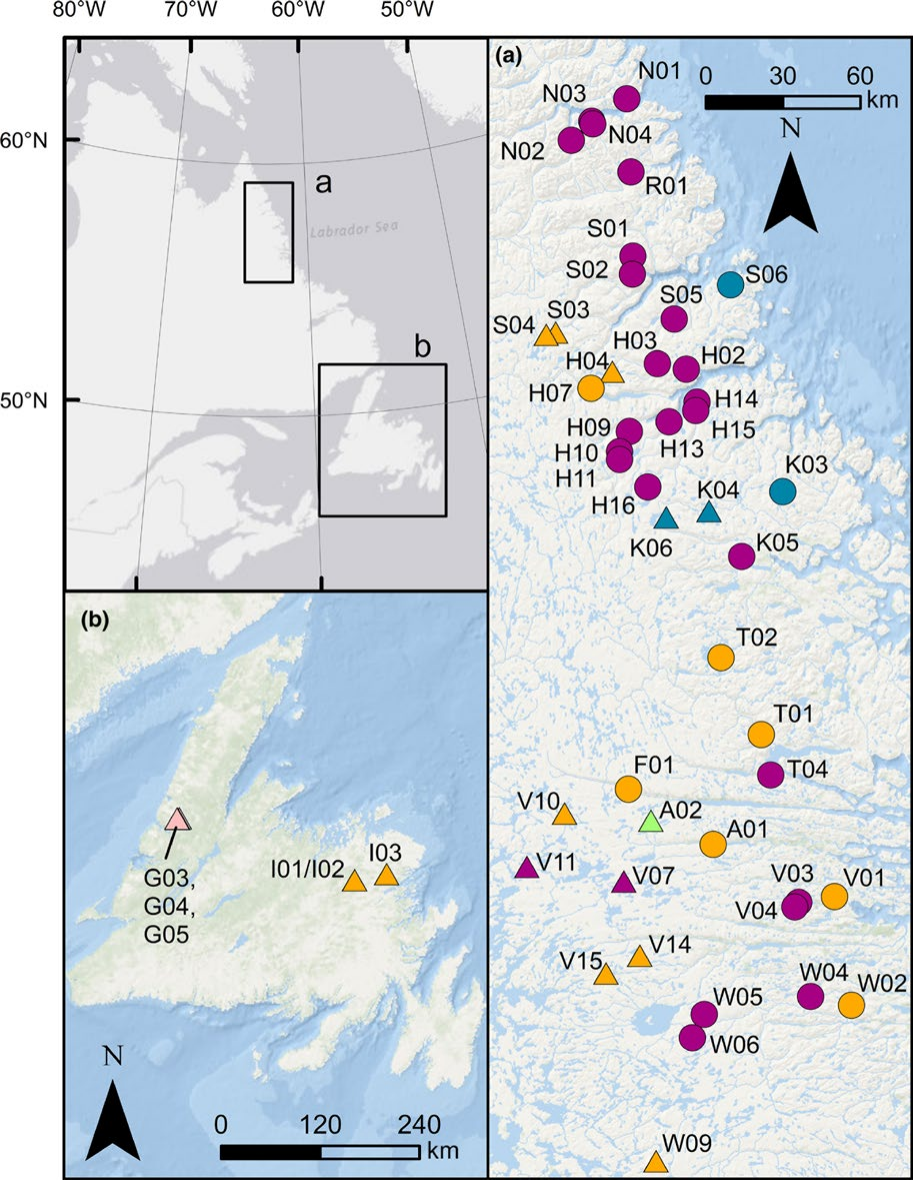
Results of SAMOVA analysis when considering all locations in (a) Labrador and (b) Newfoundland with >10 samples and taking into account geography of locations using a Delaunay matrix. Locations are colored by grouping (*K* = 5). Sea‐accessible sites are denoted by circles, and landlocked sites are denoted by triangles. Map created using ArcGIS (ESRI)

When considering all sampling locations, *F*_CT _was maximized for *K* = 6 (Figure 4). However, the difference in *F*_CT_ between *K* = 6 and *K* = 5 was small (i.e., 0.07) and a plot of *F*_CT_ versus *K* revealed that *F*_CT_ leveled off at *K* = 5 (Supporting Information Figure S1a). Given this small difference in *F*_CT,_ we report the more parsimonious results of *K* = 5. The first group contained 26 populations across Labrador with approximately equal proportions of Arctic and Atlantic lineage individuals. The second group contained only Arctic lineage individuals and comprised four locations in Labrador, three in the Okak and one in the Saglek drainage. The third group comprised only one Labrador site, A02, which contained only Acadian lineage individuals. The fourth group contained 16 populations, 14 in Labrador and two in eastern Newfoundland. This group comprised largely Atlantic lineage individuals. The fifth group contained the three landlocked lakes in western Newfoundland.

## 4 DISCUSSION

### 4.1 Extent of secondary contact

Our results indicate an extensive overlap in the contemporary ranges of the Arctic, Atlantic, and Acadian lineages in Labrador and Newfoundland. A SAMOVA detected groupings from geographically separate locations which indicates the widespread distribution of these three lineages. The two detected groupings with the highest number of locations (orange and purple groups in Figure 4) spanned the entirety of the latitudinal range sampled in Labrador. The orange group (Figure 4), which included locations with predominately Atlantic lineage fish, also included the eastern Newfoundland locations, reflecting the extensive colonization of the Atlantic lineage throughout Labrador and Newfoundland.

The Arctic and Atlantic lineage haplotypes were observed across the full latitudinal range studied in Labrador, suggesting that secondary contact has occurred at multiple times and locations among these lineages within this region. Contrary to our hypothesis, there was no association between latitude and the presence of the Arctic or Atlantic lineage in Labrador, indicating that the region in which secondary contact has occurred between these lineages is at least as extensive as our study area. Our results represent the furthest north an Atlantic haplotype has been observed in Labrador (N01, ~59°N). This observation is consistent with evidence for an incursion of Atlantic lineage nuclear DNA, but not mtDNA, in Nunavut (Moore et al., 2015). Our results also include the furthest south an Arctic lineage haplotype has been observed in the Atlantic (W06, ~55°N). It is possible that the Arctic lineage may have colonized even further south into Labrador than the range considered here. The extent of secondary contact and introgression among lineages may also be underestimated since mtDNA haplotypes reflect only maternal inheritance.

While the majority of locations in Labrador contained both the Arctic and Atlantic lineages, four populations in Labrador contained exclusively Arctic lineage samples. This group of four lakes was detected as significant by SAMOVA (blue locations in Figure 4). The absence of Atlantic lineage haplotypes (which are present in nearby populations) from these locations may have been lost through drift. Alternatively, colonization by the Atlantic lineage may have been prevented by their maladaptation to these sites (“isolation‐by‐adaptation”) or their exclusion by the previously established Arctic lineage (“isolation‐by‐colonization”) (Orsini, Vanoverbeke, Swillen, Mergeay, & Meester, 2013; Waters, 2011; Waters, Fraser, & Hewitt, 2013).

Unlike the Atlantic lineage, the Arctic lineage does not appear to have invaded Newfoundland. The absence of the Arctic lineage from Newfoundland may be due to our low sample sizes and the fewer number of locations sampled. However, our study confirmed the previous observations of the Atlantic and Acadian lineage in eastern Newfoundland (Brunner et al., 2001; Moore et al., 2015). We also demonstrated that the contemporary range of both the Atlantic and Acadian lineages extends to western Newfoundland.

Our extension of the Acadian lineage's contemporary presence into Labrador counters previous suggestions of its relatively conserved range from its putative refugium near the northeastern United States (e.g., Brunner et al., 2001; Esin & Markevich, 2018). This brings the scale of the contemporary range of the Acadian lineage in line with those observed in other char lineages.

### 4.2 Evidence for introgression

Our results here suggest extensive secondary contact but also the introgression of the Arctic, Atlantic, and Acadian lineages in Labrador and Newfoundland. Many sampled locations contained at least two glacial lineages suggesting the potential for hybridization among lineages. Furthermore, we found six sea‐accessible locations in Nachvak and Saglek fjords (N01–N04, S01–S02) contained both Arctic and Atlantic lineage individuals based on mtDNA, yet no genetic structuring was found within each of these same locations based on 11 microsatellite markers in Salisbury et al. (2018). This suggests that these lineages have fully introgressed.

Hybridization between these lineages may seem surprising given that Arctic lineage is thought to have split off from all other lineages between 716,000 and 1,432,000 years BP based on mtDNA (Moore et al., 2015). Alternatively, Esin and Markevich (2018) estimate the divergence of the Arctic lineage at 400,000–700,000 years BP during the Nebraskan–Kansan cooling. During this time, the Canadian Arctic archipelago (the putative refugium for the Arctic lineage) was separated from a refugium in the Bering Sea (Esin & Markevich, 2018). Many species have demonstrated reproductive isolation between different glacial lineages upon secondary contact within such a time scale (Bernatchez & Wilson, 1998; Hewitt, 2003). However, our results support previous research suggesting hybridization among Arctic char glacial lineages. Atlantic lineage nuclear DNA has been found in Nunavut populations of Arctic lineage individuals (Moore et al., 2015). A similar lack of a relationship between mtDNA and nuclear DNA has also been observed in three‐spine stickleback (Lescak et al., 2017). Many *Salvelinus* species are known to readily hybridize (Taylor, 2004), and there is evidence for Arctic char having hybridized with brook trout in Quebec (Bernatchez, Glémet, Wilson, & Danzmann, 1995; Glémet, Blier, & Bernatchez, 1998) and Labrador (Fraser River) (Hammar, Dempson, & Verspoor, 1991), and with lake trout in Nunavut (Wilson & Hebert, 1993) and Quebec (Wilson & Bernatchez, 1998). These hybridizations overcome a much older allopatric divergence than that among Arctic char glacial lineages. Hybridization among species does not necessitate the capacity for hybridization among intraspecific glacial lineages. However, given the relatively short duration of allopatric divergence, the lack of reproductive isolation among glacial lineages is unsurprising.

Some of the brook trout and lake trout mtDNA haplotypes detected in our samples may therefore reflect hybridization or backcrosses between these species and Arctic char. This would require further validation using nuclear markers but was beyond the scope of this study. An open area for future investigation is the degree to which genes from lake trout and brook trout have introgressed into Arctic char genomes within this region.

### 4.3 Colonization history

We detected several rare haplotypes that were previously found in other populations within each lineage's respective range allowing for insight into the origins of the three glacial lineages in this region. The ARC20 and ARC22 haplotypes we detected in Labrador were previously observed in geographically distinct locations across the high Canadian Arctic (Moore et al., 2015). The Arctic lineage may have therefore colonized Labrador multiple times from geographically distant populations. The ATL19 haplotype we observed in a single dark char morph in Gander Lake was previously observed in an unspecified morph in this lake as well as in a resident lacustrine population from Scotland (Moore et al., 2015). Lastly, the ATL31 haplotype we found in multiple anadromous populations was also found in a landlocked, Swedish population (Oleinik et al., 2017). The appearance of these Atlantic haplotypes on opposite sides of the Atlantic Ocean suggests extensive colonization throughout the Atlantic from the Atlantic refugium. While our study area demonstrates a high diversity of Atlantic lineage haplotypes, this diversity is no doubt due to our intensive sampling. Whether the Atlantic refugium was located on the western or eastern side of the Atlantic therefore requires further investigation.

### 4.4 Landlocked versus sea‐accessible locations

It was not possible to determine the order in which the glacial lineages colonized Labrador based on the lineages present in landlocked versus sea‐accessible locations. All three lineages were present in both landlocked and sea‐accessible locations in Labrador. Moore et al. (2015) suggested the Atlantic lineage had colonized the high Canadian Arctic after the Arctic lineage since some anadromous char populations contained Atlantic lineage nuclear DNA but nearby landlocked char populations demonstrated Arctic lineage nuclear DNA. Our results suggest that all three lineages may have colonized Labrador around the same time.

Though they did not share a common lineage, most landlocked populations contained a single lineage and low haplotypic diversity. This could be due to a founder‐take‐all scenario, where the lineage that first colonized a lake rapidly expanded to fill available habitat, preventing subsequent incursions from other lineages (Orsini et al., 2013; Waters, 2011; Waters et al., 2013). Also, landlocked populations are more isolated and tend to exhibit smaller effective sizes (Salisbury et al., 2018) and thus experience more drift than anadromous populations potentially leading to a greater loss of mtDNA haplotypes.

Several landlocked lakes countered this trend of reduced diversity. Landlocked lakes within the Kogaluk River system (i.e., V10, V11, V15, V16) had Arctic and Atlantic lineage char co‐occurring. Access to this watershed may have been enhanced by significant runoff from the paleolake Naskaupi, which drained through the Kogaluk between 7,500 and 6,000 years BP (Barnett & Peterson, 1964; Jansson & Kleman, 2004). Alternatively, many lakes within the Kogaluk River drainage are connected via shallow streams which could facilitate the occasional migration between lakes as it has for lake trout (McCracken, Perry, Keefe, & Ruzzante, 2013) and longnose suckers (*Catostomus catostomus*) (Salisbury, McCracken, Keefe, Perry, & Ruzzante, 2016) in this system. Migration may have countered genetic drift (Tallmon, Luikart, & Waples, 2004), maintaining both Arctic and Atlantic lineage haplotypes in these lakes. High effective sizes (Gomez‐Uchida et al., 2013) and high migration among lakes (Gomez‐Uchida et al., 2009) may have similarly countered the effects of genetic drift in landlocked populations in western Newfoundland (G02–G04) which contained both Atlantic and Acadian lineages as well as high haplotypic diversity within the Acadian lineage.

### 4.5 Glacial lineage and contemporary morph divergence in Gander Lake

Previous work has suggested that the high degree of neutral genetic differences observed between pale and dark morph char could be ascribed to differential glacial origins (Gomez‐Uchida et al., 2008). Our results, indicating that most char in Gander Lake were of the Atlantic lineage regardless of morph (aside from a single Acadian lineage pale morph char), reject this hypothesis. This suggests that the great morphological, ecological, and genetic differences between the pale and dark morph (Gomez‐Uchida et al., 2008; O'Connell & Dempson, 2002; Power, O'Connell, & Dempson, 2005) may have arisen in sympatry in Gander Lake within the last ~10,000 years since its deglaciation (Bryson et al., 1969; Dyke, 2004; Shaw et al., 2006). This is consistent with the presumed sympatric divergence of other lacustrine Arctic char morphs (Gíslason, Ferguson, Skúlason, & Snorrason, 1999; Magnusson & Ferguson, 1987; Volpe & Ferguson, 1996). The large genetic divergence among pale and dark morph char in Gander suggests substantial genetic differences can accumulate between morphs within a short period of time, potentially fueled by divergent selection (Taylor, 2004) and the relatively low effective population sizes of both pale and dark char (Gomez‐Uchida et al., 2008).

The occurrence of Atlantic and Acadian lineages in the pale morph suggests introgression of these lineages. Similar evidence for introgression among the Arctic and Atlantic lineages was found in R01 by Salisbury et al. (2018), where morphologically identified anadromous and resident char were found to be genetically differentiated by STRUCTURE but each contained both Arctic and Atlantic lineage individuals. Populations of sympatric dwarf and normal whitefish (*Coregonus clupeaformis*) in Maine have also each demonstrated both of two mtDNA haplotype groups (indicative of two glacial lineages) (Bernatchez & Dodson, 1990; Pigeon, Chouinard, & Bernatchez, 1997). These observations lead to the puzzling implication that glacial lineages have introgressed despite thousands of years of allopatric divergence yet, in some cases, their descendants have become reproductively isolated (perhaps in sympatry) and subsequently significantly diverged in the (relatively) short time since deglaciation.

### 4.6 Management implications and the utility of intensive mtDNA sampling

The likely introgression among glacial lineages in Labrador has important implications for the char fishery in Labrador. There was evidence of Arctic, Atlantic, and even Acadian lineage fish in sea‐accessible locations in the Notakwonan, Voisey, Anaktalik, Nain, and Okak drainages. These populations probably contribute to the commercial fishery stock complexes (DFO, 2001; Dempson et al., 2008). The expected introgression between lineages suggests that there is likely no need to manage them separately; however, this should be further validated by investigating the relative lineage makeup of commercially caught char.

Our results verify the utility of intensive mtDNA sampling across many populations, particularly within a secondary contact zone. This approach facilitated the detection of a number of new haplotypes for the Arctic, Atlantic, and Acadian lineages (Figures 2 and 3) as well as the detection for the first time, of the Acadian lineage within Labrador. Finally, our detection of non‐Arctic char salmonid species highlights the morphological ambiguity of salmonids, particularly as juveniles. All of the samples identified genetically as a species other than Arctic char had a median length of 40 mm (data not shown). Since species misidentification can have repercussions for the interpretation of genetic data, we therefore caution against the exclusive use of morphology in juveniles in regions where other salmonids coexist with Arctic char. The mtDNA‐based technique as used here is useful for minimizing the possibility of species misidentification in regions where other salmonid species overlap with Arctic char.

In conclusion, our results clearly demonstrate the widespread secondary contact of the Arctic, Atlantic, and Acadian glacial lineages of Arctic char throughout Labrador and Newfoundland, Canada. These three glacial lineages have likely introgressed extensively in this region. The genetic divergence in morph pairs in Ramah and Gander Lakes do not appear to be linked to glacial lineages. We demonstrate that Arctic char are an ideal model species for future investigation of secondary contact zones and the influence of historical allopatry on contemporary genetic structure and niche divergence.

## CONFLICT OF INTEREST

None declared.

## AUTHOR CONTRIBUTIONS

D.E.R., S.J.S. designed the study; all authors collected samples; G.R.M., S.J.S. completed laboratory work; S.J.S. analyzed data and led the writing.

## Data Availability

Newly identified Arctic char mtDNA D‐loop haplotypes were submitted for archival with GenBank (Accession Numbers: MK208868–MK208871, MK208875–MK208878).
