# Supplementary material for: Extensive secondary contact among three glacial lineages of Arctic Char (Salvelinus alpinus) in Labrador and Newfoundland
Source: Ecol Evol. 2019 Jan 28;9(4):2031–45. doi: 10.1002/ece3.4893 (PMC6392391; doi:10.1002/ece3.4893)
Supplement: Supplementary file 1 [file ECE3-9-2031-s001.docx]

**SUPPORTING INFORMATION**

| **Haplotype name** | **Original source** | **GenBank accession number** |
| --- | --- | --- |
| ARC19 | Alekseyev et al. 2009 | EU310899 |
| ARC20 | Moore et al. 2015 | KC907317 |
| ARC21 | Moore et al. 2015 | KC907318 |
| ARC22 | Moore et al. 2015 | KC907319 |
| ARC23 | Moore et al. 2015 | KC907320 |
| ARC24 | Moore et al. 2015 | KC907321 |
| ARC25 | Moore et al. 2015 | KC907322 |
| ARC26 | Moore et al. 2015 | KC907323 |
| ARC27 | Moore et al. 2015 | KC907324 |
| ARC28 | Moore et al. 2015 | KC907325 |
| ARC29 | Moore et al. 2015 | KC907326 |
| ARC30 | Moore et al. 2015 | KC907327 |
| ARC31 | Moore et al. 2015 | KC907328 |
| ARC32 | Moore et al. 2015 | KR011244 |
| ARC33 | Moore et al. 2015 | KR011243 |
| ARC34 | Ayers 2010 | KR011245 |
| ARC35 | New | MK208871 |
| BER10 | Alekseyev et al. 2009 | EU310900 |
| BER11 | Alekseyev et al. 2009 | EU310901 |
| BER12 | Alekseyev et al. 2009 | EU310902 |
| BER13 | Alekseyev et al. 2009 | EU310903 |
| BER14 | Moore et al. 2015 | KR011246 |
| BER15 | Moore et al. 2015 | KR011247 |
| BER16 | Ayers 2010 | KR011248 |
| BER17 | Ayers 2010 | KR011250 |
| BER18 | Ayers 2010 | KR011251 |
| BER19 | Moore et al. 2015 | KR011249 |
| HaploC | Taylor et al. 2008 | KR011254 |
| HaploY | Taylor et al. 2008 | KR011253 |
| HaploZ | Taylor et al. 2008 | KR011252 |
| SIB5 | Brunner et al. 2001 | AF298013 |
| SIB8 | Brunner et al. 2001 | AF298016 |
| SIB11 | Alekseyev et al. 2009 | EU310907 |
| SIB14 | Alekseyev et al. 2009 | EU310910 |
| SIB15 | Alekseyev et al. 2009 | EU310911 |
| SIB16 | Alekseyev et al. 2009 | EU310912 |
| SIB17 | Alekseyev et al. 2009 | EU310913 |
| SIB18 | Alekseyev et al. 2009 | EU310914 |
| SIB19 | Alekseyev et al. 2009 | EU310915 |
| SIB20 | Alekseyev et al. 2009 | EU310916 |
| SIB21 | Alekseyev et al. 2009 | EU310917 |
| SIB22 | Alekseyev et al. 2009 | EU310918 |
| **Continued on next page** | | |

**Table S1** GenBank accession numbers for all control region haplotypes used in mitochondrial analysis.

| **Table S1 continued** |  |  |
| --- | --- | --- |
| **Haplotype name** | **Original source** | **GenBank accession number** |
| SIB23 | Alekseyev et al. 2009 | EU310919 |
| SIB24 | Alekseyev et al. 2009 | EU310920 |
| SIB25 | Alekseyev et al. 2009 | EU310921 |
| SIB26 | Alekseyev et al. 2009 | EU310922 |
| SIB29 | Alekseyev et al. 2009 | EU310925 |
| SIB30 | Alekseyev et al. 2009 | EU310926 |
| SIB31 | Moore et al. 2015 | KR011255 |
| ATL1 | Brunner et al. 2001 | AF297991 |
| ATL4 | Brunner et al. 2001 | AF297994 |
| ATL19 | Moore et al. 2015 | KR011258 |
| ATL20 | Moore et al. 2015 | KR011261 |
| ATL21 | Moore et al. 2015 | KR011257 |
| ATL22 | Moore et al. 2015 | KR011256 |
| ATL23 | Salisbury et al. 2018 | MK208872 |
| ATL24 | Salisbury et al. 2018 | MK208873 |
| ATL25 | Salisbury et al. 2018 | MK208874 |
| ATL26 | New | MK208875 |
| ATL28 | New | MK208876 |
| ATL29 | New | MK208877 |
| ATL31 | New | MK208878 |
| ACD9 | Alekseyev et al. 2009 | EU310898 |
| ACD10 | Moore et al. 2015 | KR011259 |
| ACD11 | Moore et al. 2015 | KR011260 |
| ACD12 | New | MK208868 |
| ACD13 | New | MK208869 |
| ACD14 | New | MK208870 |
| *S. fontinalis* | Keskin, E. (GenBank only) | HQ167705 |
| *S. namaycush* | Taylor et al. 2008 | KT362731 |
| *S. salmo* | Oleinik, A.G. (GenBank only) | KY122303 |
|  | | |

**Table S2** *Salvelinus alpinus* D-loop haplotypes observed in sampling locations.

| **Site** | **ACD9** | **ACD11** | **ACD12** | **ACD13** | **ACD14** | **ATL01** | **ATL04+**  **ATL31** | **ATL04** | **ATL31** | **ATL01 or**  **ATL04** | **ATL19** | **ATL23** | **ATL24** | **ATL25** | **ATL26** | **ATL28** | **ATL29** | **ARC19 +**  **ARC19 or ARC24** | **ARC19** | **ARC19 or ARC24** | **ARC20** | **ARC22** | **ARC35** |
| --- | --- | --- | --- | --- | --- | --- | --- | --- | --- | --- | --- | --- | --- | --- | --- | --- | --- | --- | --- | --- | --- | --- | --- |
| **N01** |  |  |  |  |  | 9 |  |  |  |  |  |  |  |  |  |  | 1 | 5 |  | 5 |  |  |  |
| **N02** |  |  |  |  |  | 5 |  |  |  |  |  |  |  |  |  |  |  | 19 | 1 | 18 |  |  |  |
| **N03** |  |  |  |  |  | 10 | 2 | 1 | 1 |  |  |  |  |  |  |  |  | 12 | 1 | 11 |  |  |  |
| **N04** |  |  |  |  |  | 7 |  |  |  |  |  |  |  |  |  |  |  | 12 | 1 | 11 |  |  |  |
| **R01** |  |  |  |  |  | 31 | 1 |  | 1 |  |  |  |  |  |  |  |  | 15 | 1 | 14 |  | 1 |  |
| **S01** |  |  |  |  |  | 12 |  |  |  |  |  |  |  |  |  |  |  | 13 | 1 | 12 |  |  |  |
| **S02** |  |  |  |  |  | 9 | 1 |  | 1 |  |  | 1 |  |  |  |  |  | 13 | 1 | 12 |  |  |  |
| **S03** |  |  |  |  |  |  |  |  |  |  |  | 24 |  |  |  |  |  |  |  |  |  |  |  |
| **S04** |  |  |  |  |  |  |  |  |  |  |  | 22 | 1 | 1 |  |  |  |  |  |  |  |  |  |
| **S05** |  |  |  |  |  | 3 | 1 | 1 |  |  |  |  |  |  |  |  |  | 10 |  | 10 |  |  |  |
| **S06** |  |  |  |  |  |  |  |  |  |  |  |  |  |  |  |  |  | 23 | 1 | 22 |  |  |  |
| **H02** |  |  |  |  |  | 11 | 4 | 3 | 1 |  |  |  |  |  |  |  |  | 8 | 1 | 7 |  |  |  |
| **H03** |  |  |  |  |  | 5 | 1 |  | 1 |  |  |  |  |  |  |  |  | 10 | 2 | 8 |  |  |  |
| **H04** |  |  |  |  |  | 24 |  |  |  |  |  |  |  |  |  |  |  |  |  |  |  |  |  |
| **H05** |  |  |  |  |  |  | 1 | 1 |  |  |  |  |  |  |  |  |  | 3 |  | 3 |  |  |  |
| **H07** |  |  |  |  |  | 18 |  |  |  |  |  |  |  |  |  |  |  | 6 | 1 | 5 |  |  |  |
| **H09** |  |  |  |  |  | 10 |  |  |  |  |  |  |  |  |  |  |  | 14 | 1 | 13 |  |  |  |
| **H10** |  |  |  |  |  | 7 |  |  |  |  |  |  |  |  |  |  |  | 15 |  | 15 |  |  |  |
| **H11** |  |  |  |  |  | 7 | 1 |  | 1 |  |  |  |  |  |  |  |  | 13 | 1 | 12 |  |  |  |
| **H12** |  |  |  |  |  | 1 |  |  |  |  |  |  |  |  |  |  |  | 2 |  | 2 |  |  |  |
| **H13** |  |  |  |  |  | 10 |  |  |  |  |  |  |  |  |  |  |  | 14 | 1 | 13 |  |  |  |
| **H14** |  |  |  |  |  | 7 | 1 |  | 1 |  |  |  |  |  |  |  |  | 15 | 1 | 14 |  |  |  |
| **H15** |  |  |  |  |  | 10 |  |  |  |  |  |  |  |  |  |  |  | 10 | 1 | 9 |  |  |  |
| **H16** |  |  |  |  |  | 8 |  |  |  |  |  |  |  |  |  |  |  | 10 |  | 10 |  |  |  |
| **K01** |  |  |  |  |  | 3 |  |  |  |  |  |  |  |  |  |  |  | 4 | 1 | 3 |  |  |  |
| **K02** |  |  |  |  |  |  |  |  |  |  |  |  |  |  |  |  |  | 6 | 1 | 5 |  |  |  |
| **K03** |  |  |  |  |  |  |  |  |  |  |  |  |  |  |  |  |  | 24 | 1 | 23 |  |  |  |
| **K04** |  |  |  |  |  |  |  |  |  |  |  |  |  |  |  |  |  | 20 |  | 20 | 1 |  |  |
| **K05** |  |  |  |  |  | 11 | 1 |  | 1 |  |  |  |  |  |  |  |  | 12 | 1 | 11 |  |  |  |
| **K06** |  |  |  |  |  |  |  |  |  |  |  |  |  |  |  |  |  | 24 |  | 24 |  |  |  |
| **T01** |  |  |  |  |  | 17 | 4 | 3 | 1 |  |  |  |  |  |  | 1 |  | 1 | 1 |  |  |  | 1 |
| **T02** |  |  |  |  |  | 25 |  |  |  |  |  |  |  |  |  | 1 |  |  |  |  |  |  |  |
| **T03** |  |  |  |  |  |  |  |  |  |  |  |  |  |  |  |  |  | 7 |  | 7 |  |  |  |
| **T04** |  |  |  |  |  | 11 |  |  |  |  |  |  |  |  |  |  |  | 10 | 1 | 9 |  |  |  |
| **T05** |  |  |  |  |  | 1 |  |  |  |  |  |  |  |  |  |  |  |  |  |  |  |  |  |
| **F01** |  |  |  |  |  | 15 | 7 | 7 |  |  |  |  |  |  |  | 1 |  | 1 |  | 1 |  |  |  |
| **A01** |  |  |  |  |  | 20 |  |  |  |  |  |  |  |  | 1 |  |  | 3 | 1 | 2 |  |  |  |
| **Continued on next page** | | | | | | |  |  |  |  |  |  |  |  |  |  |  |  |  |  |  |  |  |
| **Table S2 Continued** | | | | | | | |  |  |  |  |  |  |  |  |  |  |  |  |  |  |  |  |
| **Site** | **ACD9** | **ACD11** | **ACD12** | **ACD13** | **ACD14** | **ATL01** | **ATL04+**  **ATL31** | **ATL04** | **ATL31** | **ATL01 or**  **ATL04** | **ATL19** | **ATL23** | **ATL24** | **ATL25** | **ATL26** | **ATL28** | **ATL29** | **ARC19 +**  **ARC19 or ARC24** | **ARC19** | **ARC19 or ARC24** | **ARC20** | **ARC22** | **ARC35** |
| **A02** | 22 |  |  |  |  |  |  |  |  |  |  |  |  |  |  |  |  |  |  |  |  |  |  |
| **V01** |  |  |  |  |  | 18 |  |  |  |  |  |  |  |  |  |  |  | 4 | 1 | 3 |  |  |  |
| **V02** |  |  |  |  |  | 1 |  |  |  |  |  |  |  |  |  |  |  | 1 | 1 |  |  |  |  |
| **V03** |  |  |  |  |  | 9 |  |  |  |  |  |  |  |  |  |  |  | 9 |  | 9 |  |  |  |
| **V04** |  |  |  |  |  | 5 |  |  |  |  |  |  |  |  |  |  |  | 9 | 1 | 8 |  |  |  |
| **V05** |  |  |  |  |  |  |  |  |  |  |  |  |  |  |  |  |  | 1 | 1 |  |  |  |  |
| **V06** |  |  |  |  |  |  |  |  |  |  |  |  |  |  |  |  |  | 5 | 1 | 4 |  |  |  |
| **V07** |  |  |  |  |  | 1 |  |  |  | 1 |  |  |  |  |  |  |  | 12 |  | 12 |  |  |  |
| **V09** |  |  |  |  |  | 9 |  |  |  |  |  |  |  |  |  |  |  |  |  |  |  |  |  |
| **V10** |  |  |  |  |  | 11 |  |  |  |  |  |  |  |  |  |  |  | 1 |  | 1 |  |  |  |
| **V11** |  |  |  |  |  | 11 |  |  |  |  |  |  |  |  |  |  |  | 12 |  | 12 |  |  |  |
| **V13** |  |  |  |  |  |  |  |  |  |  |  |  |  |  |  |  |  | 6 |  | 6 |  |  |  |
| **V14** |  |  |  |  |  | 15 |  |  |  | 2 |  |  |  |  |  |  |  |  |  |  |  |  |  |
| **V15** |  |  |  |  |  | 9 |  |  |  | 2 |  |  |  |  |  |  |  | 2 |  | 2 |  |  |  |
| **V16** |  |  |  |  |  | 1 |  |  |  |  |  |  |  |  |  |  |  | 6 |  | 6 |  |  |  |
| **W01** |  |  |  |  |  | 3 |  |  |  |  |  |  |  |  |  |  |  |  |  |  |  |  |  |
| **W02** |  |  |  |  |  | 12 |  |  |  |  |  |  |  |  |  |  |  |  |  |  |  |  |  |
| **W03** | 1 |  |  |  |  | 6 |  |  |  |  |  |  |  |  |  |  |  | 1 | 1 |  |  |  |  |
| **W04** |  |  |  |  |  | 7 |  |  |  |  |  |  |  |  |  |  |  | 9 |  | 9 |  |  |  |
| **W05** |  |  |  |  |  | 2 |  |  |  |  |  |  |  |  |  |  |  | 10 |  | 10 |  |  |  |
| **W06** |  |  |  |  |  | 1 |  |  |  |  |  |  |  |  |  |  |  | 11 |  | 11 |  |  |  |
| **W09** |  |  |  |  |  | 16 |  |  |  |  |  |  |  |  |  |  |  |  |  |  |  |  |  |
| **G01** | 4 |  |  |  |  |  |  |  |  |  |  |  |  |  |  |  |  |  |  |  |  |  |  |
| **G02** | 5 |  |  |  |  | 1 |  |  |  |  |  |  |  |  |  |  |  |  |  |  |  |  |  |
| **G03** | 11 | 3 |  |  | 1 | 1 |  |  |  |  |  |  |  |  |  |  |  |  |  |  |  |  |  |
| **G04** | 7 | 2 | 1 |  |  | 2 |  |  |  |  |  |  |  |  |  |  |  |  |  |  |  |  |  |
| **G05** | 11 | 6 | 3 | 1 |  |  |  |  |  |  |  |  |  |  |  |  |  |  |  |  |  |  |  |
| **G06** |  |  |  |  |  |  |  |  |  |  |  |  |  |  |  |  |  |  |  |  |  |  |  |
| **I01** |  |  |  |  |  | 21 |  |  |  |  | 1 |  |  |  |  |  |  |  |  |  |  |  |  |
| **I02** | 1 |  |  |  |  | 20 |  |  |  |  |  |  |  |  |  |  |  |  |  |  |  |  |  |
| **I03** |  |  |  |  |  | 24 |  |  |  |  |  |  |  |  |  |  |  |  |  |  |  |  |  |
| **Total** | **62** | **11** | **4** | **1** | **1** | **513** | **25** | **16** | **9** | **5** | **1** | **47** | **1** | **1** | **1** | **3** | **1** | **453** | **29** | **424** | **1** | **1** | **1** |

**SAMOVA RESULTS**

When geography was not considered in the SAMOVA model for all sampling locations, F_CT_ was maximized for K = 4 (Fig.S1a). The first grouping was identical to the first grouping when considering geography but did not contain the populations V07 or W06 (Fig.S2b). These two sampling locations were composed of predominately of Arctic lineage individuals and were instead grouped with the 4 Labrador populations containing only Arctic lineage individuals (S06, K03, K04, K06). The third group contained A02 with the populations from the west coast of Newfoundland. The fourth group was identical to the fourth group when geography was considered.

SAMOVA analyses of only the Labrador populations found F_CT_ was maximized for K = 6 for the geography-dependent model. However, the difference in F_CT_ between K = 6 and K = 4 was 0.08 and plots of F_CT_ versus K revealed that F_CT_ leveled off at K = 4(Fig.S1b). Given this small difference in F_CT_ we report the more parsimonious results of K = 4 here. The first group contained 27 locations and was nearly identical to that of the full model considering geography but also included H07 (Fig.S2c). The second group contained the four all-Arctic lineage populations. The third group contained 13 locations containing predominately Atlantic lineage samples. The final group was composed of A02.

SAMOVA analyses of only the Labrador populations found F_CT_ was maximized for K = 5 for the geography-independent model (Fig.S1b). However, the difference in F_CT_ between K = 5 and K = 4 was 0.07 and plots of F_CT_ versus K revealed that F_CT_ leveled off at K = 4 (Fig.S1b). Given this small difference in F_CT_ we report the more parsimonious results of K = 4 here. The groupings were identical to the geography-independent model except W06 was grouped with the four all-Arctic populations (Fig.S2d).

SAMOVA analyses of only the Newfoundland populations found K = 2 to maximize F_CT_ using both the geography-dependent and geography-independent models (Fig.S1c). Both models separated the east coast populations from the west coast populations (Fig.S2e,f).

**CT**

a) b)

c)

**CT**

Geography

No Geography

**Fig.S1** F_CT_ versus K-value for SAMOVA analyses of a) all sampling locations, b) only Labrador sampling locations, c) only Newfoundland sampling locations. Filled circles indicate SAMOVA analyses for which geography was taken into account using a Delaunay matrix, open circles indicate SAMOVA analyses for which geography was not taken into account.


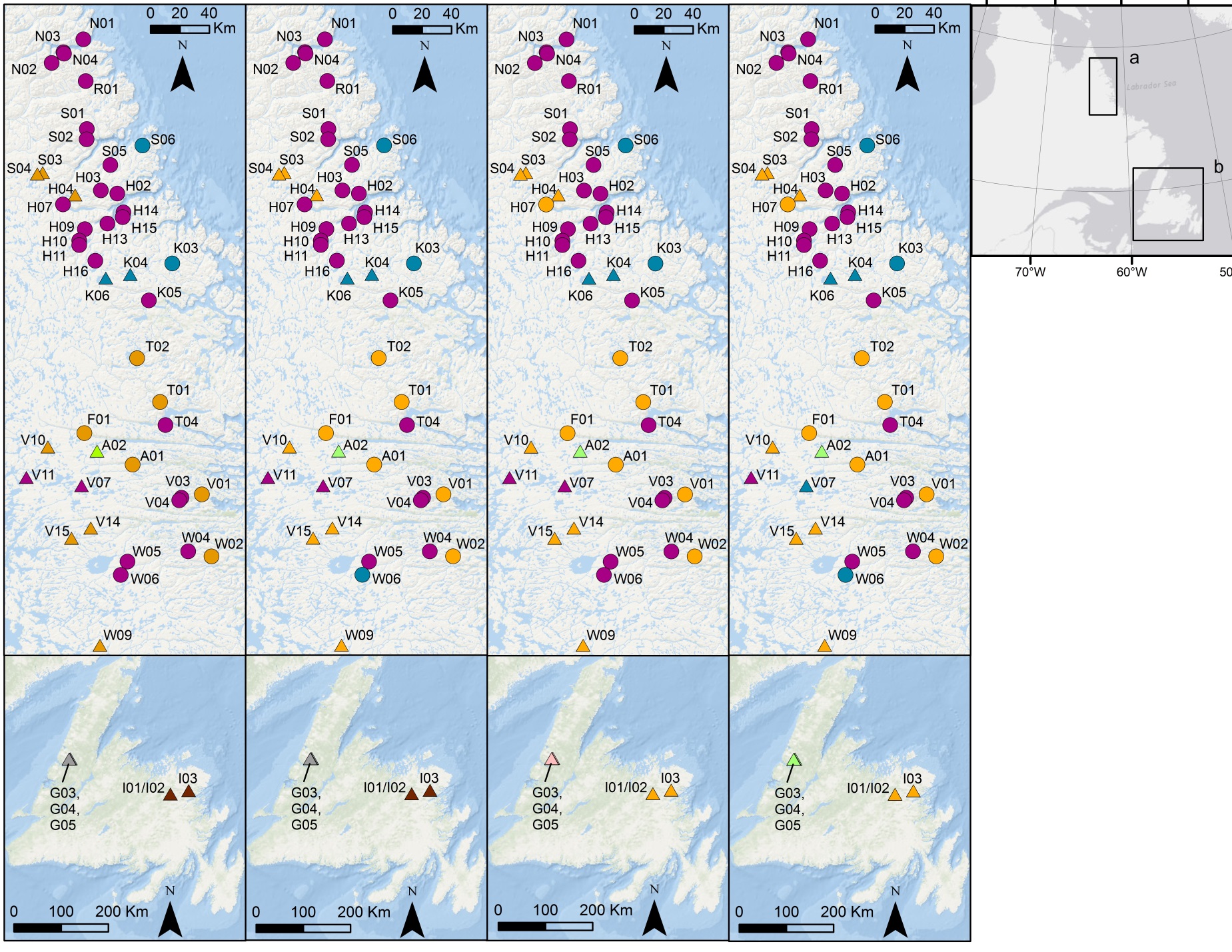

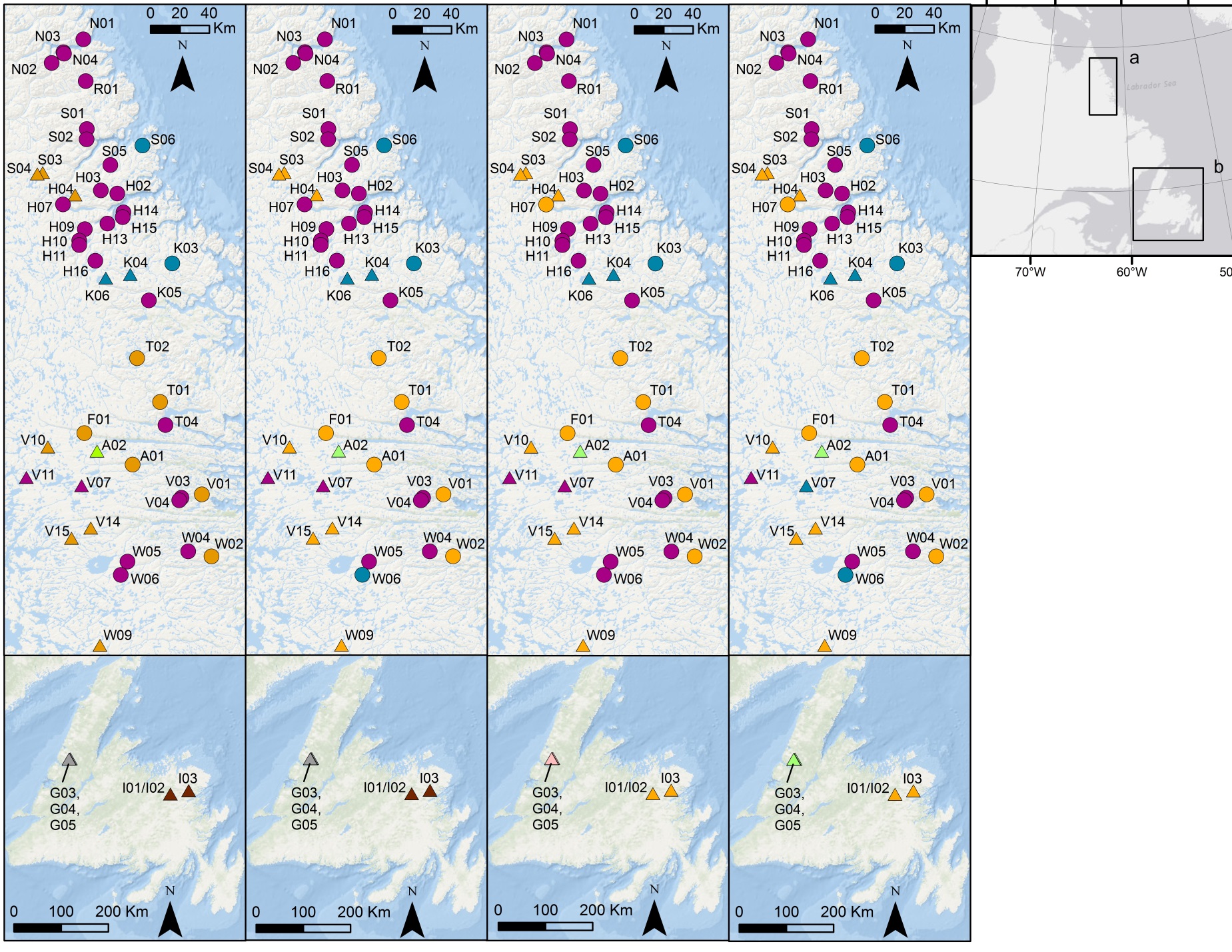


a) b) c) d)

e) f)

**Fig.S2** Results of SAMOVA analyses for all sampling locations a) with (K = 5) and b) without (K = 4) the consideration of geography; for only Labrador sampling locations c) with (K = 4) and d) without (K = 4) the consideration of geography; for only Newfoundland sampling locations e) with (K = 2) and f) without (K = 2) the consideration of geography. Locations are coloured by grouping. Sea-accessible sites are denoted by circles, landlocked sites are denoted by triangles. Map created using ArcGIS (ESRI).

**References**

Alekseyev, S.S., Bajno, R., Gordeeva, N.V., Reist, J.D., Power, M., Kirillov, A.F., Samusenok, V.P., & Matveev, A.N. (2009). Phylogeographic patterns and sympatric differentiation in the Arctic charr *Salvelinus alpinus* (L.) complex from Siberia as revealed by mitochondrial DNA sequence analysis, with special reference to Transbaikalia. Journal of Fish Biology. *75*, 368–392.

Ayers, S.D. (2010). A review of the species status of the Angayukaksurak charr (*Salvelinus anaktuvudensis*) of Northern Alaska: perspectives from molecular and morphological data. MSc Thesis, University of Alaska Fairbanks, Fairbanks.

Brunner, P.C., Douglas, M.R., Osinov, A., Wilson, C.C., & Bernatchez, L. (2001). Holarctic phylogeography of Arctic charr (*Salvelinus alpinus L.*) inferred from mitochondrial DNA sequences. *Evolution*, 55, 573–586.

Moore, J.S., Bajno, R., Reist, J.D., & Taylor, E.B. (2015). Post-glacial recolonization of the North American Arctic by Arctic char (*Salvelinus alpinus*): genetic evidence of multiple northern refugia and hybridization between glacial lineages. *Journal of biogeography*, *42*(11), 2089-2100.

Salisbury, S.J., Booker, C., McCracken, G.R., Knight, T., Keefe, D., Perry, R., and Ruzzante, D. E. (2018). Genetic divergence among and within Arctic char (*Salvelinus alpinus*) populations inhabiting landlocked and sea-accessible sites in Labrador, Canada. *Canadian Journal of Fisheries and Aquatic Sciences, 75*(8), 1256-1269.

Taylor, E.B., Lowery, E., Lilliestråle, A., Elz, A., & Quinn, T.P. (2008). Genetic analysis of sympatric char populations in western Alaska: Arctic char (*Salvelinus alpinus*) and Dolly Varden (*Salvelinus malma*) are not two sides of the same coin. *Journal of Evolutionary Biology*, *21*(6), 1609-1625.
